# Metabolic regulation of GLP-1 and PC1/3 in pancreatic α-cell line

**DOI:** 10.1371/journal.pone.0187836

**Published:** 2017-11-09

**Authors:** Veronica Sancho, Giuseppe Daniele, Daniela Lucchesi, Roberto Lupi, Annamaria Ciccarone, Giuseppe Penno, Cristina Bianchi, Angela Dardano, Roberto Miccoli, Stefano Del Prato

**Affiliations:** 1 Department of Clinical and Experimental Medicine, Section of Diabetes and Metabolic Diseases, University of Pisa – Cisanello Hospital, Pisa, Italy; 2 Section of Diabetes and Metabolic Diseases, Azienda Ospedaliero–Universitaria Pisana, Cisanello Hospital, Pisa, Italy; University of Ulster, UNITED KINGDOM

## Abstract

**Background and aims:**

An intra-islet incretin system has been recently suggested to operate through modulation of the expression and activity of proconvertase 1/3 and 2 (PC1/3, PC2) in pancreatic alpha-cell accounting for local release of GLP-1. Little is known, whether this alpha-cell activity can be affected by the metabolic alterations occurring in type 2 diabetes, such as hyperglycemia, hyperlipidemia or hyperglucagonemia.

**Materials and methods:**

AlphaTC1/6 cells from a mice pancreatic cell line were incubated in the presence of two glucose (G) concentration (5.5 and 16.7 mM) for 16 h with or without free fatty acid, IL6 or glucagon. GLP-1 secretion was measured by ELISA and expression of PC1/3 and PC2 by RT-PCR and western blot; cell viability was determined by MTT method, Reactive Oxygen Species generation (ROS) by H_2_DCFDA fluorescence and apoptosis by Annexin staining and Propidium Iodine (PI) fluorescence.

**Results:**

Upon 16.7G incubation, GLP-1 secretion (total and active) was significantly increased in parallel with a significant increment in PC1/3 expression, a slight increase in cell viability and ROS generation and by a decrement in PC2 expression with no change in cell apoptosis. When cells were incubated at 5.5mM glucose with FFA, also an increment in GLP-1 secretion and PC1/3 expression was observed together an increment in ROS generation, a decrement in cell viability, and a modest increment in apoptosis. When incubated with 16.7mM glucose with FFA, the increment in GLP-1 secretion was reduced to basal, accompanied by an increment in apoptosis and ROS generation. This was also observed with IL-6, but in this case, no modification in ROS generation or apoptosis was observed when compared to 16.7mM glucose. The presence of glucagon did not modify any of the parameters studied.

**Conclusion:**

These data suggest that under hyperglycemic, hyperlipidemia or inflammatory conditions, alpha cells can increase expression PC1/3 and activate GLP-1 secretion, which may contribute protecting both alpha and beta-cells from glucose and lipotoxicity, while this effect seems to be lost in the presence of both pathophysiological conditions.

## Introduction

Glucagon like-peptide–1 (GLP-1), one of the main incretin hormones, is secreted by the entero-endocrine L cell of the intestine [1] after nutrient ingestion [2]. The hormone is the post-transductional product of the preproglucagon gene. Once cleaved to proglucagon the protein is processed by the enzyme proconvertase 1/3 (PC1/3) with the formation moieties corresponding, among others, to GLP-1 and GLP-2 [1,2].

The pancreatic alpha cell is the main source of circulating glucagon, which also is derived from proglucagon via cleavage by proconvertase 2 [1]. More recently, it has been proposed that GLP-1 can be secreted by the pancreatic alpha cell through the activity of PC1/3 leading to the hypothesis of an intra-islet incretinergic system that may exert direct paracrine effect on the beta cell [3].

By immunofluorescence technique, Marchetti *et al* [4] were able to confirm the co-presence of GLP-1 and PC1/3 in the alpha cells of human pancreatic islets isolated from non-diabetic and type 2 diabetic cadaveric donors. They also showed that PC1/3 expression and GLP-1 secretion were more pronounced in the diabetic islets [4]. In cultured rat islets as well as pancreatic alpha cell lines, high glucose was shown to increase GLP-1 secretion and PC1/3 expression along with a reduction of glucagon secretion [3]. Moreover, in isolated human and mice islets, interleukin-6 (IL-6) was shown to elicit an increase of GLP-1 and glucagon secretion as well as PC1/3 expression [5]. Similar effects have been observed in pregnant and neonatal mouse [6] and db/db [7] and ob/ob mice [8] progressing toward overt diabetes. In these conditions a shift from glucagon- to GLP-1-positive cells was found along with an increment in cells with double positivity for GLP-1 and PC1/3. On the contrary, no modification of GLP-1 expression was reported in the NOD type 1 diabetic mouse [7].

Altogether these observations support the hypothesis that synthesis and release of glucagon and GLP-1 in from pancreatic alpha cell can be modulated by a number of stimuli. Therefore, this study aimed at exploring whether glucagon and GLP-1 secretion and related PC2 (proconvertase 2) or PC1/3 expression could be modulated by metabolic alterations typically occurring in type 2 diabetes, such as hyperglycemia, hyperlipidemia, hyperglucagonemia, and subclinical inflammation.

## Matherial and methods

### Cell culture and experimental design

The alpha-TC1/6 pancreatic alpha cell line was obtained from American Type Culture Collection (ATCC^®^ CRL-2934^™^, USA). Cells were cultured in DMEM, 4 mmol/l L-glutamine, 2 g/l glucose, 1.5 g/l sodium bicarbonate, 15 mmol/l Hepes, 0.02% BSA, 0.1 mmol/l non-essential amino acids, 100 IU/mL penicillin and 100 μg/mL streptomycin and 10% FBS, in CO_2_/O_2_ (95%/5%) at 37°C and passaged weekly medium changed every other day. Cells at 20–25 passages were used in all the experiments described below.

Once at confluence cells were trypsinized and seeded at 50,000 cells/well density for assessment of viability, ROS production, and apoptosis. For mRNA expression 2x10^6^ cell/well were used and 4x10^6^ cell/well for protein assessment. Before each experiment cells were deprived from serum, and incubated in the presence of glucose (16.7 mM), FFA (0.5 mM), glucagon (500 pg/ml) or IL-6 (200 ng/ml) for the assessment of parameters of interest. Glucose and IL-6 were added directly to the medium, while FFA were conjugated to 5% BSA before being added in. The same BSA concentration was ensured for the other experimental conditions as well. Glucagon, total, and active GLP-1 levels in the medium were measured by ELISA method (R&D Systems, Adbingdon, UK and Merck Millipore, Darmstadt, Germany) and values normalized by protein content. All samples were collected in the presence of Ile-Pro-Ile, DPP-IV inhibitor, at final concentration of 0.1 mg/ml as previusly described [9]. Reactive Oxigen Species (ROS) generation was determined by fluorescence of the specific intracellular probe CM-H_2_DCFDA as measured in a Cary Eclipse Fluorimeter at 494/527 excitation/emission nm. Cell viability was determined by the Thiazolyl Blue Tetrazolium Blue method and Apotosis by the Annexin V-FITC Apoptosis Detection Sigma-Aldrich Kit, where Annexin V-FICT binding to phosphstidilserine is related to apoptosis and Propidium Iodine binding to DNA is related to necrosis and apoptosis.

After cells were incubated for 16 h in the presence of 5.5mM or 16.7mM, and glucagon, or FFA or IL-6; total mRNA was extracted with Tryzol (Invitrogen) and RNA yield determined by spectrophotometric absorbance at 260 nm; determination of PC1/3, PC2 and Gcg mRNA expression was performed by Real Time PCR carried out using Applied Biosystem Step One Plus (PE Applied Biosystem, Warrington, UK) using the TaqMan gene expression assays, the TaqMan Universal PCR Master Mix and specific primers. Data were normalized by the housekeeping 18S mRNA. All samples were assayed in triplicate and results presented as average fold-increase compared to control.

Proteins were extracted upon cell lysis with CelLytic M and a protease inhibitor cocktail (Sigma-Aldrich, St. Louise, MO). Protein concentration was determined for each sample by Bradford reagent and absorbance measured at 595 nm wavelength on a Thermo Multiscan Ex Plate spectrophotometer. Protein expression was detected by SDS-PAGE and Western Blotting, as described previously [10].

### Reagents

Most reagents were purchased from Sigma-Aldrich (St. Louise, MO). PC1/3, PC2 or Actin first antobodies and all HRP-conjugated secondary antibodies were purchased from Santa Cruz Biotechnologies (Dallas, TX). Pre-developed TaqMan gene expression assays, TaqMan Universal PCR Master Mix, CM-H2DCFDA, TaqMan Universal PCR Master Mix and Trizol were from Life Technologies (Grand Island, NY). iScript cDNA Synthesis kit, 4–20% Tris-Glycine gels precast gels and Trans-blot Tranfer Nitrocellulose membrane 0.20 μm were purchased from BioRad Laboratories Inc. (Hercules, CA). GLP-1 total and active form ELISA Kit were from Merck Millipore (Darmstadt, Germany). Glucagon quantikine ELISA Kit was from R&D Systems (Adbingdon, UK).

### Statistical analysis

All experiments were performed at least 4 times. Data are presented as mean±SEM. Unpaired Student's t-test (two-tailed) was used for single comparisons, while one-way ANOVA was carried out for multiple comparisons. P values < 0.05 were considered statistically significant.

## Results

### Cell viability and apoptosis

**Fig 1:**
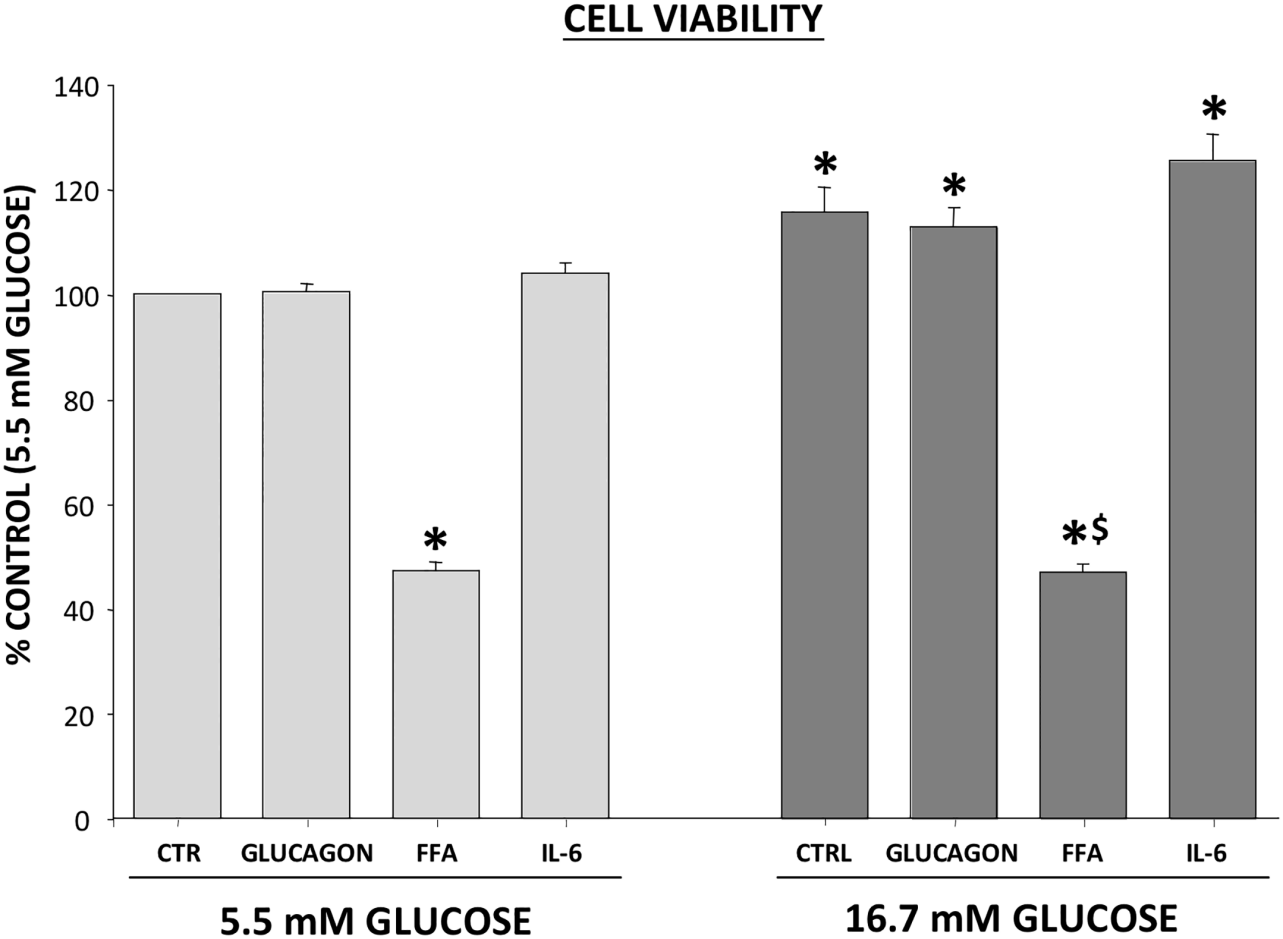
Effect of metabolic/hormonal pertubation on cell viability. Cells were incubated for 16 h in the presence of 5.5mM or 16.7mM, in the additional presence of glucagon, FFA and IL-6 and cell viability was determined by MTT method. Results are % of control (cells incubated in 5.5mM glucose) and expressed as MEAN±SEM, n = 4. *p<0.05 compared to 5.5mM glucose control; $p<0.05 compare to 16.7mM glucose control.

Alpha-TC1/6 cells viability was not affected, if any increased, by 16h exposure to high glucose (16.7 mM) as compared with incubation with 5.5 mM glucose (Fig 1).

Adding glucagon (500 pg/ml) or IL-6 (200 ng/ml) did not modify cell viability, while FFA (0.5 mM) caused a significant reduction in cell viability (47±2%, *p =* 0.001; Fig 1). Glucagon, FFA and IL-6 did not affect annexin FICT binding, a marker of apoptosis, when added to the medium at 5.5 mM glucose. However, apoptosis increased when FFA were added to medium containing 16.7 mM glucose (152±11%, *p =* 0.000). This finding was confirmed by a 42% increase (*p<*0.001) of propidium iodine incorporation into cell nuclei (S1 Fig).

### Effect of high glucose on glucagon and GLP-1 synthesis and secretion

**Fig 2:**
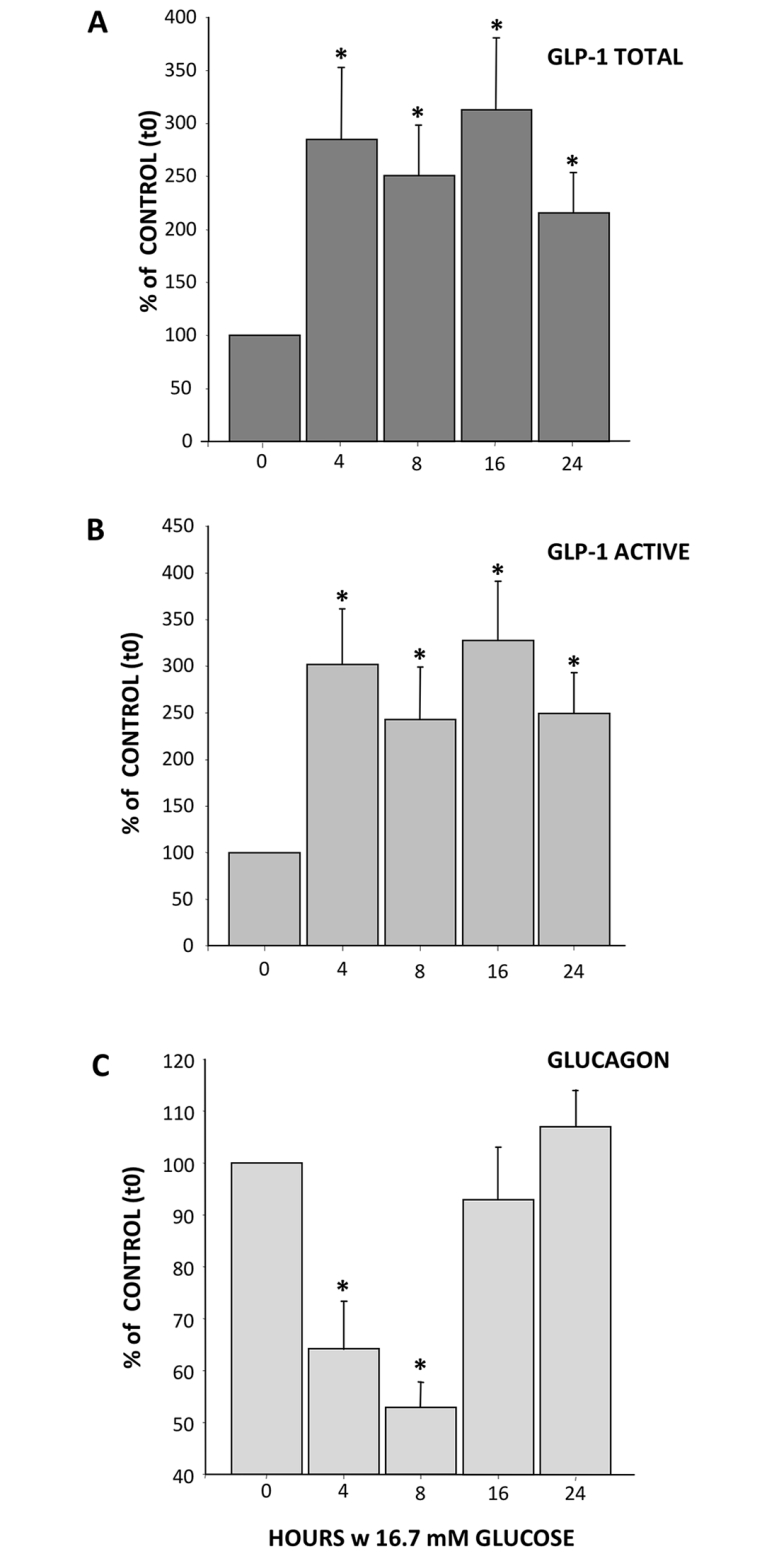
Effect of high glucose on glucagon and GLP-1 secretion. Cells were incubated with 16.7mM glucose and hormone concentration in the medium determined by ELISA method. Panel A: Total GLP-1 secretion; Panel B: Active GLP-1 secretion and Panel C: Glucagon secretion. Results are % of control and expressed as MEAN±SEM, n = 4. *p<0.05 compared to control.

Alpha-TC1/6 cells were exposed to high glucose (16.7 mM) for up to 24 h (Fig 2).

Both total and active GLP-1 content increased from 4h through 24h (Total GLP-1: +285±67% and +216±37% vs. baseline, respectively, *p<*0.05; active GLP-1: +302±59% and +249±43%, respectively, *p =* 0.003) reaching a peak effect at 16h (Total GLP-1: +313±59%, *p =* 0.004; active GLP-1: +327±63%, *p =* 0.001; Fig 2A and 2B). Conversely, glucagon secretion decreased significantly after 4h until 12h (4h: 64±9%, 6h: 50±5%, 8h: 53±5% and 12h: 72±9%, respectively) to return to baseline condition at 16h (Fig 2C).

Changes in GLP-1 concentration overall were paralleled by changes in PC1/3 expression, which increased at 8h (3.03±1.02 folds) reaching a zenith at 16h (5.00±0.30 folds; *p =* 0.006) to remain higher than in control cultures up to 24h (4.66±2.40 folds; *p =* 0.001). As the maximum GLP-1 secretion and PC1/3 expression were obtained after 16h incubation, this incubation time was used in all subsequent experiments.

### Effect of metabolic/hormonal perturbation on glucagon and GLP-1 synthesis and secretion

At 5.5 mM glucose total GLP-1 was modestly increased (p = NS) in response to glucagon, FFA and IL-6 exposure. On the contrary, it increased significantly at high glucose (130±9%, *p =* 0.001), it remained unchanged in presence of high glucagon and strongly lowered by FFA and IL-6 exposure (128±17%, 107±6% p = 0.054 *vs* HG and 101±7%, *p* = 0.019 vs HG). Similarly, active GLP-1 increased in a non-significant manner in response to glucagon at low glucose, while a significant increase was elicited by the FFA and IL-6 (173±21%, *p<*0.02 and 156±12%, *p<*0.05, respectively). The glucagon increase elicited by IL-6 and HG was associated with a significant increment in Gcg expression (1.61±0.12 times *vs* Control *p =* 0.0059) (S2 Fig).

**Fig 3:**
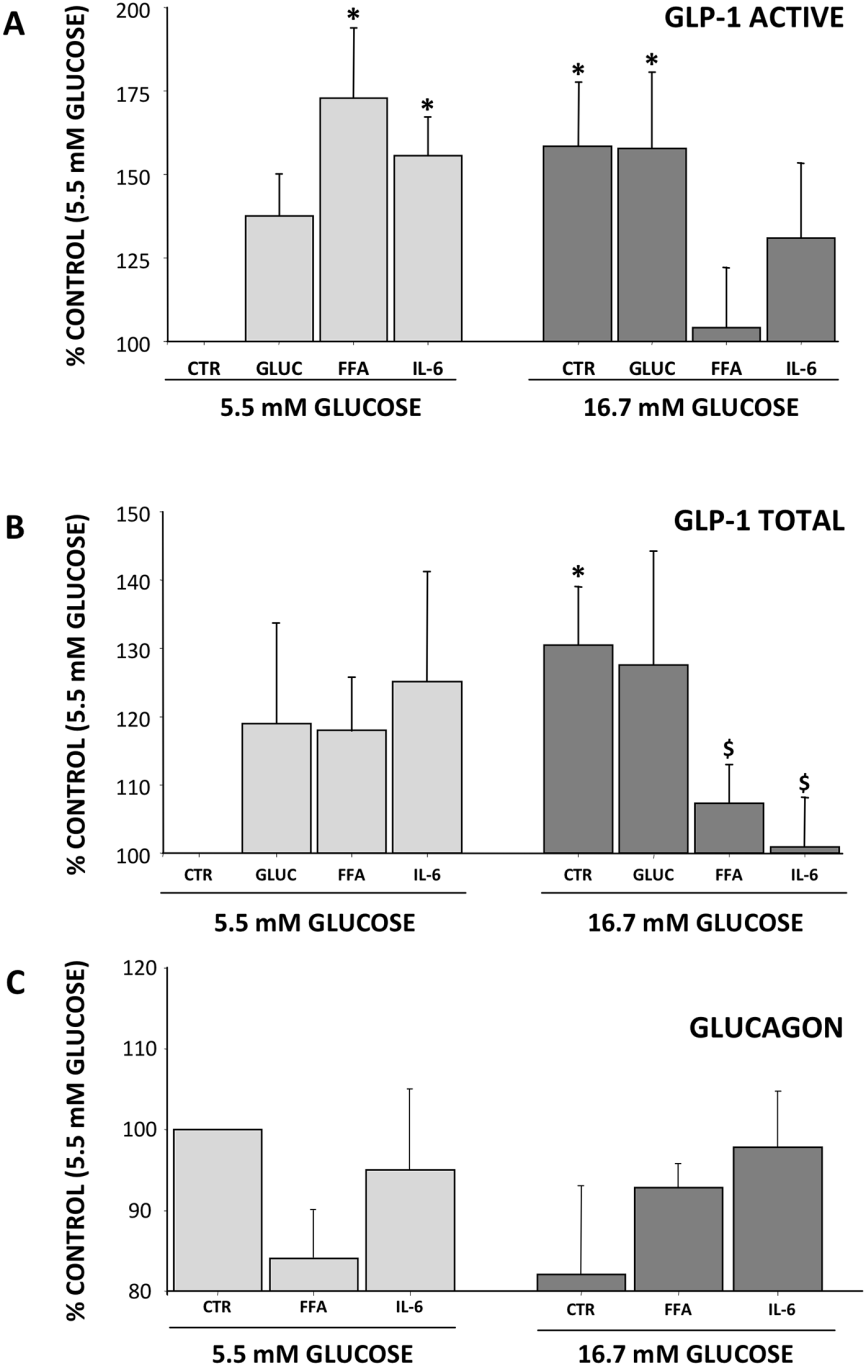
Effect of metabolic/hormonal pertubation on GLP-1 secretion. Cells were incubated for 16 h in the presence of 5.5mM or 16.7mM, in the additional presence of glucagon, FFA and IL-6; hormone secretion at medium was determined by ELISA method using specific kits. Panel A: GLP-1 Active, Panel B: GLP-1 Total and Panel C: Glucagon. Results are % of control (cells incubated in 5.5mM glucose) and expressed as MEAN±SEM, n = 4. *p<0.05 compared to 5.5mM glucose control; $p<0.05 compare to FFA (5.5mM glucose).

Glucagon did not interfere with the increase in GLP-1 observed with high glucose, while active GLP-1 release was strongly reduced in response to FFA and IL-6 (Fig 3A and 3B).

Glucagon secretion was lower, although not significant, at 16.7 mM than at 5.5 mM glucose and glucagon levels in the medium were not affected by IL-6, irrespective of glucose concentration (Fig 3C).

**Fig 4:**
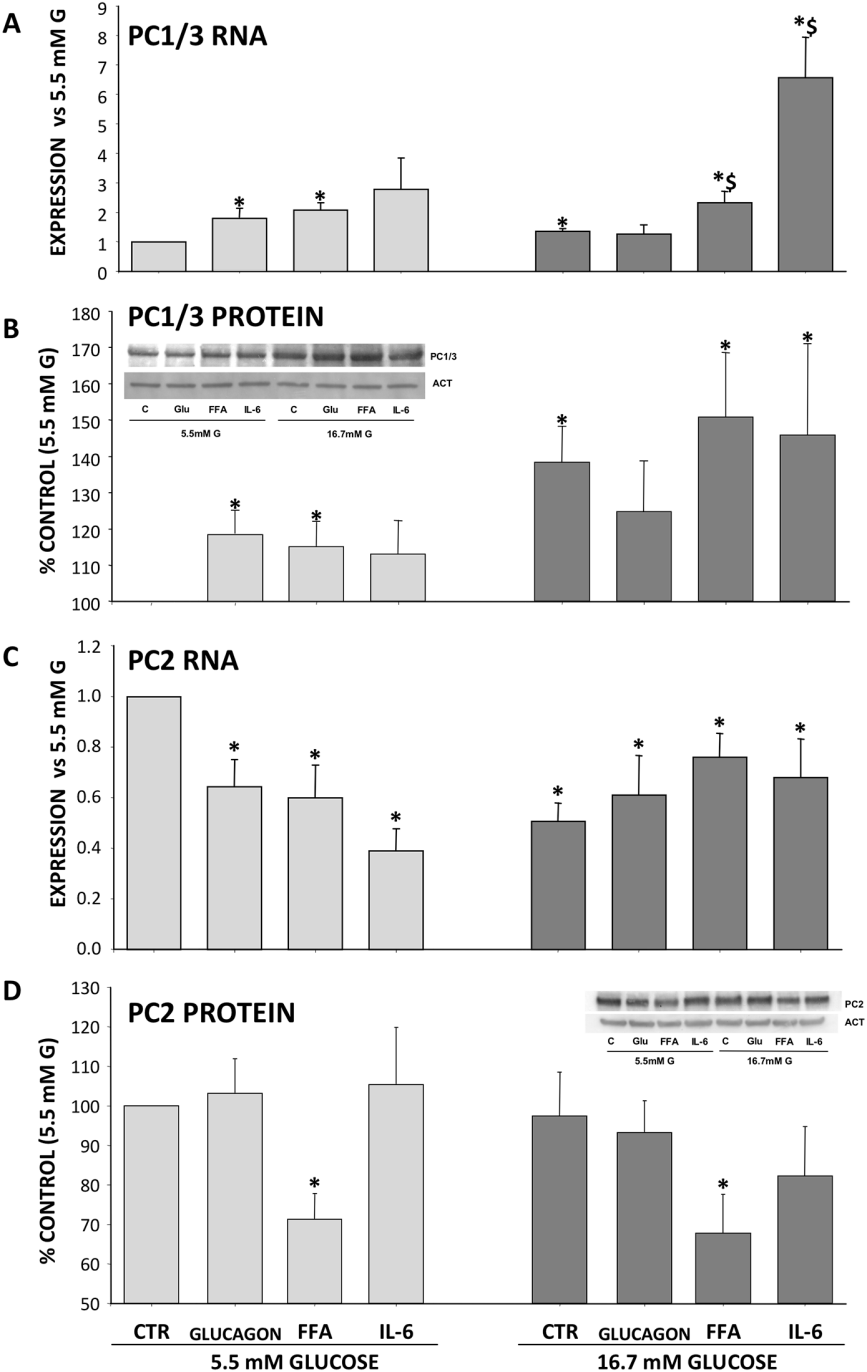
Effect of metabolic/hormonal pertubation on PC1/3 and PC2 expression. Cells were incubated for 16 h in the presence of 5.5mM or 16.7mM, in the additional presence of glucagon, FFA and IL-6, total mRNA and protein were extracted and PC1/3 and PC2 expression were determined by Real Time PCR using specific primers and SDS-PAGE and Western Blotting. Panel A: PC1/3 RNA, Panel B: PC1/3 protein, Panel C: PC2 RNA and Panel D: PC2 protein. Results are Times vs control (5.5mM glucose) and % of control (5.5mM glucose) and expressed as MEAN±SEM, n = 5–7. *p<0.05 compared to control; $p<0.05 compare to control (16.7mM glucose).

In parallel, we studied PC1/3 and PC2 mRNA expression and relative protein content (Fig 4).

At 5.5 mM glucose, glucagon, FFA and IL-6 increased PC1/3 mRNA expression (mRNA: 1.81±0.31% *p =* 0.041, 2.07±0.24%, and 2.78±1.04% *p =* 0.006, respectively, all *p*<0.05 or less) as well as protein level (119±8%, 117±4% *p<*0.03, and 124±7%, respectively, all *p*<0.05 or less). In presence of high glucose concentrations, IL-6 (6.56±1.37%, *p =* 0.032) but not glucagon nor FFA increased expression of PC1/3 mRNA. Although PC1/3 protein levels at high glucose concentration were significantly higher that those observed with 5.5 mM glucose (138±10% *p =* 0.010), the presence of IL-6 did not further modify the proconvertase expression (protein: 146±25% *p<*0.02 *vs* LG, NS *vs* HG) (Fig 4A and 4B).

The reduction in glucagon secretion was associated with a concomitant reduction of PC2 mRNA expression. While a reduction in PC2 protein expression occurred after cell exposure to FFA both at 5.5 or 16.7 mM glucose (71±7% and 78±10%, *p<*0.05 *vs* LG alone) (Fig 4C and 4D).

### ROS production

In order to gain information on potential mechanisms mediating glucagon/GLP-1 secretion in response to metabolic and hormonal stimuli we assessed ROS production (S3 Fig).

At low glucose, FFA but glucagon and IL-6, caused a significant increase of ROS production (132±8%, *p =* 0.001). Conversely, high glucose produced a significant increase of ROS production (120±7%, *p =* 0.008) that remained unaltered with high glucagon and IL-6 (117±8% and 110±5%, NS *vs* HG alone) and further enhanced by FFA (139±9%, *p =* 0.017).

## Discussion

In this study we have evaluated the reciprocal modulation of glucagon and GLP-1 from alpha cells in response to metabolic and hormonal conditions that are commonly seen in type 2 diabetes. To this purpose we have used alpha-TC1/6 cells. These cells are derived from a neoplastic cell-line generated in transgenic mice expressing the SV40 large T antigen oncogene under the control of the rat pre-proglucagon promoter. Although the parental alpha-TC1 cell line produces glucagon and considerable quantities of insulin and pre-proinsulin mRNA, the clonal line (clone 6) we have used is terminally differentiated and produces glucagon but not insulin nor it expresses pre-proinsulin mRNA. These cells offer the advantage, over those isolated from pancreatic islets, of being more homogeneous as cell population and they have been previously used to study glucagon secretion and gene expression [11–13]. On the other hand, these results cannot be extrapolated to the physiologic condition where alpha-cells lay in strict contact with the other endocrine cells of the panreatic islet.

By using this alpha cell model we have confirmed that a number of metabolic stimuli can modulate glucagon/GLP-1 synthesis and release in a reciprocal manner. Thus, when exposed to high glucose concentration, GLP-1 release was enhanced and glucagon release was reduced. A similar response has been observed in human and rat islets [3, 13].

Increased GLP-1 release occurred with a parallel increase of PC1/3 RNA expression. Similar results have been reported in isolated rat islet [3] using even higher glucose concentrations. In a murine model of type 2 diabetes (db/db mouse) [7] and in pregnant and neonatal mice [6] a shift from glucagon positive to GLP-1 positive cells was observed. This shift was paralleled by an increment in cells double positive for GLP-1 and PC1/3.

Upon confirmation that alpha-TC1/6 cells release GLP-1, we have explored whether this release could be modulated by other metabolic alterations commonly seen in type 2 diabetes. This was done to both at low (5.5 mM) and high (16.7 mM) glucose concentration.

Fat ingestion is known to elicit GLP-1 release *in vivo* [14, 15]. This response in part is due to a direct effect of the fatty acids on L cell [16]. Our study shows a similar effect on alpha-TC1/6 cells: in the presence of normal glucose concentration, FFA induced an increase in PC1/3 expression and GLP-1 release much in agreement with previous findings in NCI-H716 cells, fetal rat intestinal cultured cells, or GLUTag cells. In these cells both oleic and palmitic acid triggered GLP-1 release [17–19]. We have not investigated the mechanism responsible for this effect though activation of Grp40 and Grp120 is plausible. These receptors have been detected on the pancreatic alpha cell and Grp120 agonists have been shown to increase of GLP-1 secretion and PC1/3 expression [3, 20]. Given the deleterious impact of increased FFA on the beta cell it may be tempting to hypothesize that the increased GLP-1 secretion by the alpha cell may exert a protective paracrine effect as suggested by the work of Thyssen *et al* [6] and O’Malley *et al* [7]. However, the combination of hyperglycemia and FFA may be deleterious to the alpha cell in line with the glucolipotoxicity effect reported for the beta-cell. Our results, indeed, suggest that in alpha-TC1/6 cells glucolipotoxicity increases ROS production, apoptosis rate, and reduces viability.

Type 2 diabetes is associated with subclinical inflammation as indicated by the increase of circulating proinflammatory cytokines as such as TNF-α, IL-6, IL-1ß or IL-18 [21–23]. Among these, IL-6 has been proposed as an incretinogenic factor and a modulator of alpha cell mass [5, 24, 25]. Rats injected with IL-6 or GLUTag cells exposed to the cytokine, showed increased circulating GLP-1 levels, increased expression of GLP-1 mRNA along with increased PC1/3 expression [5]. In keeping with these findings, incubation of alpha-TC1/6 cells in the presence of IL-6 at low glucose (5.5mM) induced an increment in GLP-1 secretion and PC1/3 expression, with no changes in glucagon secretion, PC2 expression, cell viability, ROS generation or apoptosis rate. Such a stimulatory effect, however, was lost in a high glucose ambient, in spite of increased PC1/3 expression.

The decrement in the ratio glucagon/GLP-1 by IL-6 in the alpha cell under hyperglycemic conditions, if taking place during type 2 diabetes pathogenesis in the islet enviorement could produce a reduction in beta cell apoptosis, a better insulin response to glucose by the beta cell, and increment in the alpha cell mass and maybe a higher rate of transdifferentiation from alpha to beta cell.

Glucagon levels are inappropriately elevated in subjects with type 2 diabetes. Therefore, we have also investigated whether high glucagon concentration could have an effect on GLP-1 secretion from alpha-TC1/6 cells. To the best of our knowledge this has not been determined before. Our results clearly show that the exposure of pancreatic alpha cells to high glucagon did not alter any of the parameters tested both at low and high glucose levels. This is at variance with a potential direct effect of GLP-1. Piro *et al* have shown that exposure of alpha-TC1/6 cells to GLP-1 is associated with a reduction of glucagon secretion, activation of the cAMP/MAPK/Pax6 pathway, and increased Gcg gene, and protein expression along with a significant increase in PC1/3 protein expression, GLP-1 intracellular content, and GLP-1 secretion.

In summary, our data suggest that metabolic/hormonal conditions characterizing type 2 diabetes can exert a modulatory effect on the reciprocal regulation of synthesis and secretion of glucagon and GLP-1 in alpha-TC1/6 cells resulting in increased expression of PC1/3 expression and GLP-1 release. However, such effects appear to be hampered by concomitant high glucose levels, a condition associated with marked oxidative stress.

## Supporting information

S1 Fig Effect of metabolic/hormonal pertubation on cell apoptosis. Cells were incubated for 16 h in the presence of 5.5mM or 16.7mM, in the additional presence of glucagon, FFA and IL-6 and cell apoptosis was determined by fluorescence emission of annexin-V binding to phosphatidilserine and propidium iodine binding to DNA. Results are % of control (cells incubated in 5.5mM glucose) and expressed as MEAN±SEM, n = 8. *p<0.05 compared to 5.5mM glucose control.
(TIFF)

S2 Fig Effect of metabolic/hormonal pertubation on Gcg expression. Cells were incubated for 16 h in the presence of 5.5mM or 16.7mM, in the additional presence of glucagon, FFA and IL-6, total mRNA was extracted and Gcg expression were determined by Real Time PCR using specific primers Results are Times vs control (5.5mM glucose) and expressed as MEAN±SEM, n = 4. *p<0.05 compared to control; $p<0.05 compare to control (16.7mM glucose).
(TIFF)

S3 Fig Effect of metabolic/hormonal pertubation on ROS production. Cells were incubated for 16 h in the presence of 5.5mM or 16.7mM, in the additional presence of glucagon, FFA and IL-6, ROS production was determined by fluorescence of the specific intracellular probe CM-H_2_DCFDA as measured in a Cary Eclipse Fluorimeter at 494/527 excitation/emission nm Results are % of control (cells incubated in 5.5mM glucose) and expressed as MEAN±SEM, n = 4. *p<0.05 compared to 5.5mM glucose control; $p<0.05 compare to 16.7mM glucose control.
(TIFF)
