# Supplementary figures and images for: Metabolic regulation of GLP-1 and PC1/3 in pancreatic α-cell line
Source: PLoS One. 2017 Nov 9;12(11):e0187836. doi: 10.1371/journal.pone.0187836 (PMC5679617; doi:10.1371/journal.pone.0187836)

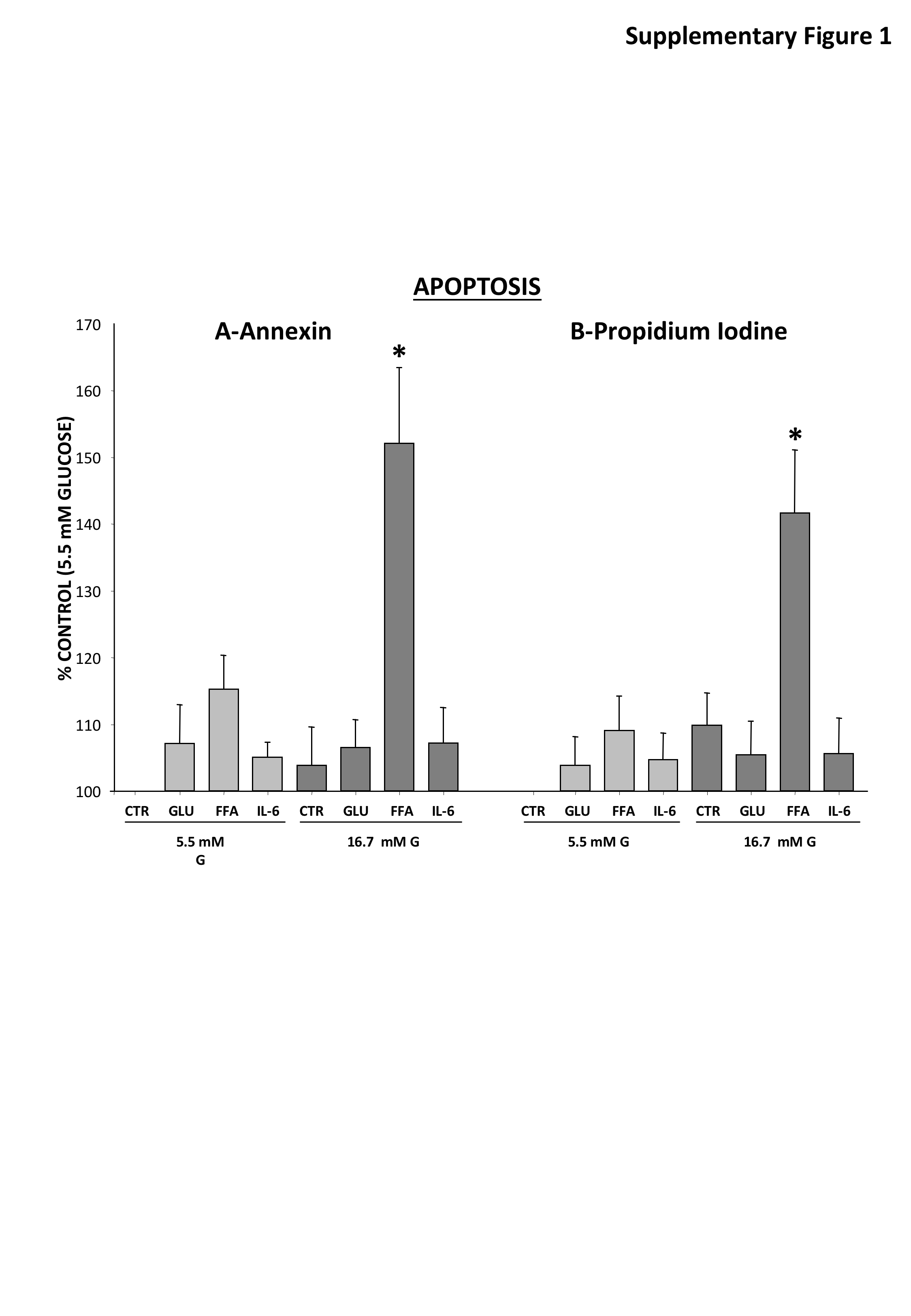

Supplement: S1 Fig — Cells were incubated for 16 h in the presence of 5.5mM or 16.7mM, in the additional presence of glucagon, FFA and IL-6 and cell apoptosis was determined by fluorescence emission of annexin-V binding to phosphatidilserine and propidium iodine binding to DNA. Results are % of control (cells incubated in 5.5mM glucose) and expressed as MEAN±SEM, n = 8. *p<0.05 compared to 5.5mM glucose control. (TIFF) [file pone.0187836.s001.tiff]

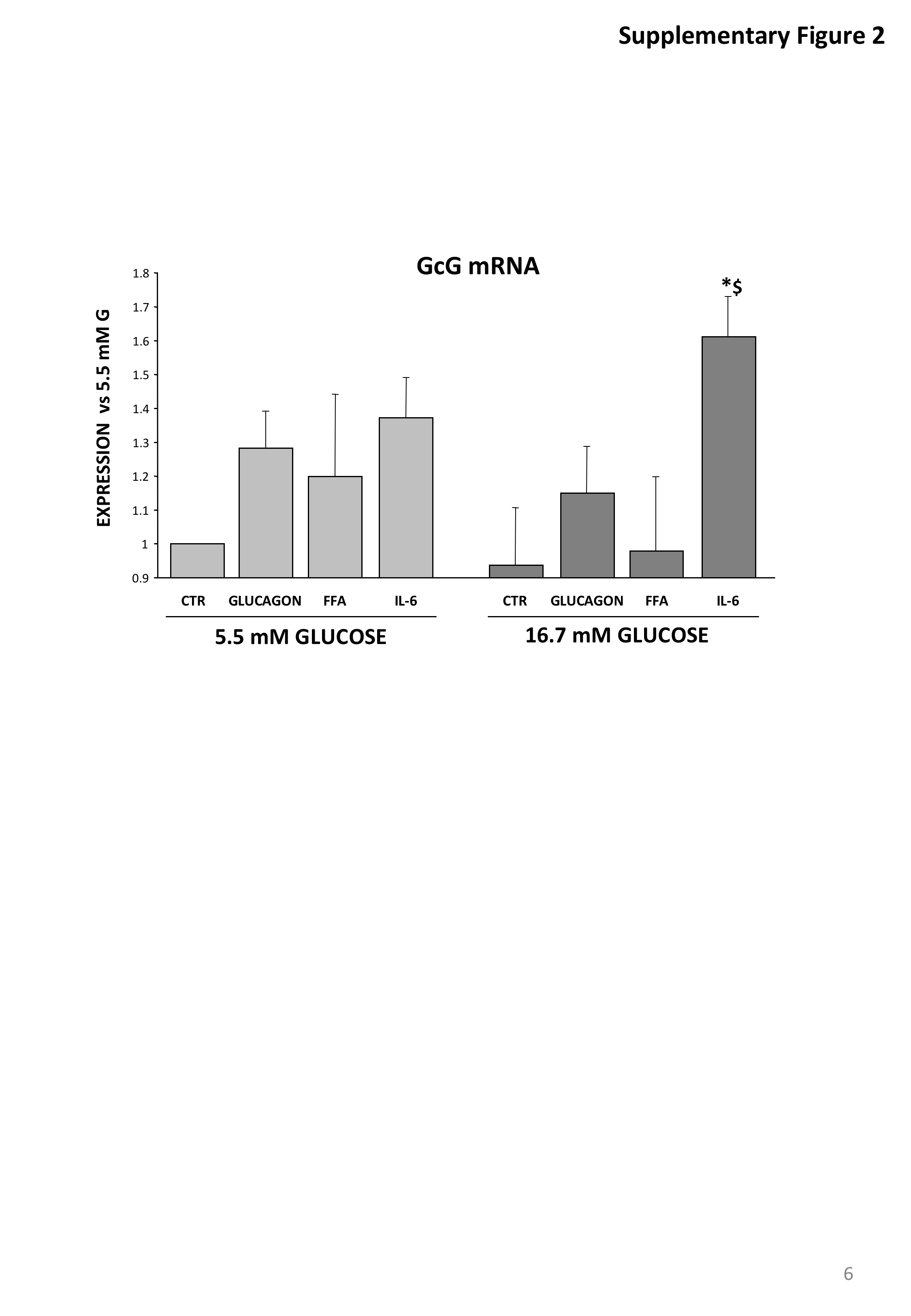

Supplement: S2 Fig — Cells were incubated for 16 h in the presence of 5.5mM or 16.7mM, in the additional presence of glucagon, FFA and IL-6, total mRNA was extracted and Gcg expression were determined by Real Time PCR using specific primers Results are Times vs control (5.5mM glucose) and expressed as MEAN±SEM, n = 4. *p<0.05 compared to control; $p<0.05 compare to control (16.7mM glucose). (TIFF) [file pone.0187836.s002.tiff]

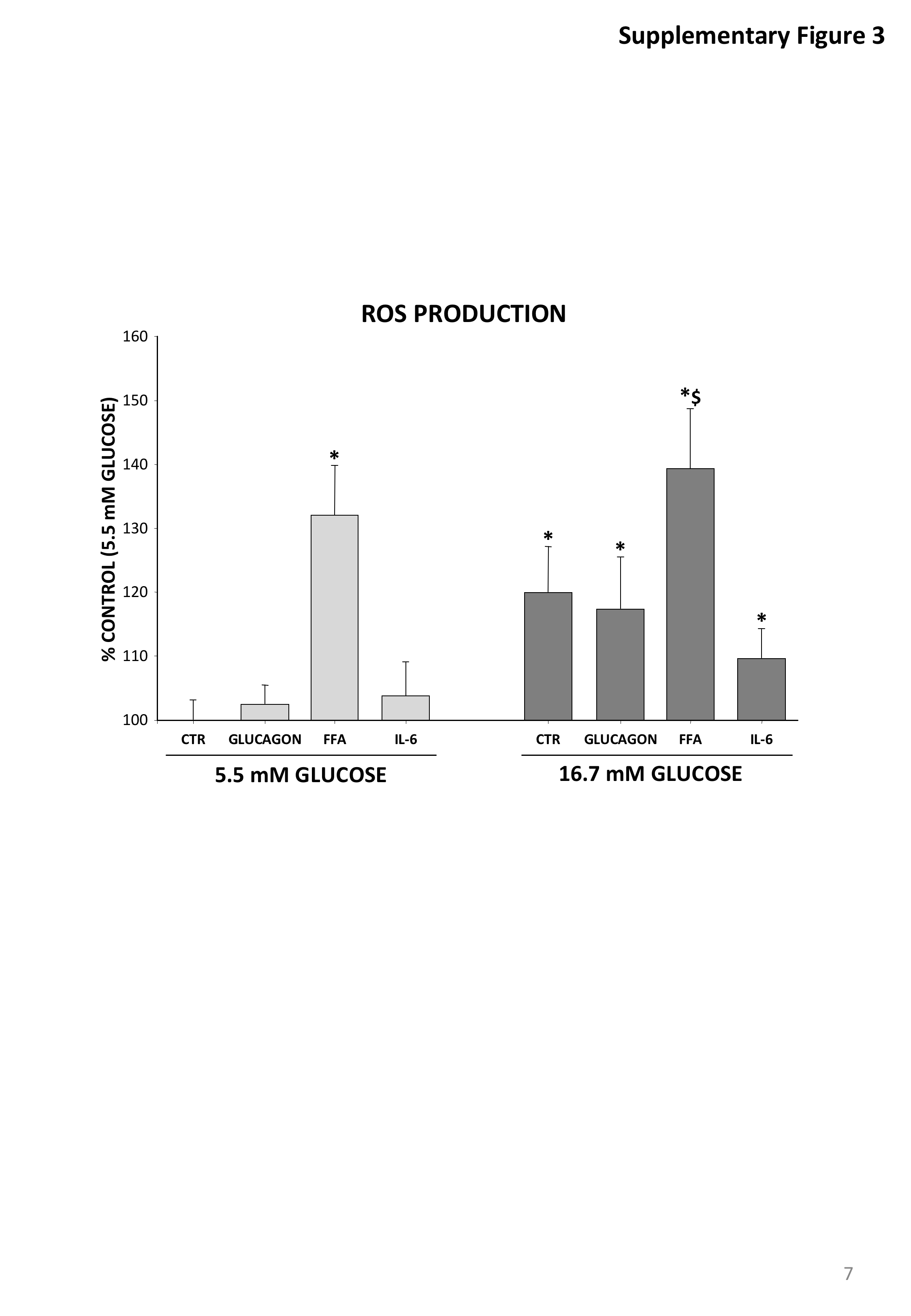

Supplement: S3 Fig — Cells were incubated for 16 h in the presence of 5.5mM or 16.7mM, in the additional presence of glucagon, FFA and IL-6, ROS production was determined by fluorescence of the specific intracellular probe CM-H2DCFDA as measured in a Cary Eclipse Fluorimeter at 494/527 excitation/emission nm Results are % of control (cells incubated in 5.5mM glucose) and expressed as MEAN±SEM, n = 4. *p<0.05 compared to 5.5mM glucose control; $p<0.05 compare to 16.7mM glucose control. (TIFF) [file pone.0187836.s003.tiff]
